# A Framework for Two-class Classification of Pulmonary Tuberculosis using Artificial Intelligence

**DOI:** 10.2174/0115734056343819250307040956

**Published:** 2025-04-17

**Authors:** Akansha Nayyar, Rahul Shrivastava, Shruti Jain

**Affiliations:** 1 Department of BT & BI, Jaypee University of Information Technology, Solan, H.P., India; 2 Department of Electronics and Communication Engineering, Jaypee University of Information Technology, Solan, India

**Keywords:** *Mycobacterium tuberculosis*, Chest X-ray images, Machine learning techniques, Gray Level Difference Statistics, Shape features, Edge features

## Abstract

**Aim:**

The study investigates the creation and assessment of Machine Learning (ML) models using different classifiers such as Support Vector Machine (SVM), logistic regression, decision tree, k-nearest neighbour (kNN), and Artificial Neural Network (ANN) for the automated identification of tuberculosis (TB) from chest X-ray (CXR) images.

**Background:**

As a persistent worldwide health concern, TB requires early detection for effective treatment and control of the infection. The differential diagnosis of TB is a challenge, even for experienced radiologists. With the use of automated processing of CXR images which are reasonable and frequently used for TB diagnosis, employing Artificial Intelligence (AI) techniques provides novel possibilities.

**Objective:**

The objective of the study was to identify respiratory disorders, radiologists devote a lot of time reviewing each of the CXR images. As such, they can identify the type of disease using automated methods based on AI algorithms. This work advances the diagnosis of TB via machine learning, which may result in early treatment options and enhanced outcomes for patients.

**Methods:**

The disease was classified using distinct parameters like edge, shape, and Gray Level Difference Statistics (GLDS) on splitting of the dataset at 70:30 and 80:20.

**Results:**

It was observed that authors attained 93.5% accuracy using SVM with linear kernel for a 70:30 data split considering hybrid parameters. The comparison was made considering different feature extraction techniques, different dataset splitting, existing work, and another dataset.

**Conclusion:**

The designed model using SVM, decision tree, kNN, ANN, and logistic regression was compared using other state-of-the-art techniques, other datasets, different feature extraction techniques, and different splitting of data. AI has great promise for enhancing tuberculosis detection, which will ultimately lead to an earlier diagnosis and improved disease management.

## 1 INTRODUCTION

*Mycobacterium tuberculosis*, an aerobic bacterium, is primarily responsible for causing tuberculosis (TB) disease and belongs to the genus *Mycobacteria* [1, 2]. The genus *Mycobacteria* includes *Non-tuberculous Mycobacteria (NTM)* [3] and *Mycobacterium tuberculosis* Complex (MTBC), among others. *M. tuberculosis* belongs to the MTBC group. The members of MTBC are genetically similar and cause TB or other related diseases. The World Health Organization (WHO) Global TB Report 2024 highlights 10.8 million new cases of tuberculosis, up from 10.6 million in 2022, with over two-thirds concentrated in eight high-burden countries, including India [4]. Approximately two-thirds of estimated incident cases globally were found in eight countries from 30 high-burden countries, with India having the highest number of instances in 2023. These countries constituted 87% of all projected incident cases worldwide. TB remains a significant global health problem, particularly in developing countries and among immunocompromised individuals (such as those with HIV/AIDS). Tuberculosis affects various organs of the human body, based on this, TB disease is classified into numerous forms, including skeletal TB, which extends from the lungs to the bones; miliary TB, which affects the lungs and bone marrow but can also infect other regions of the body such as the brain or even the heart; and liver TB, which is regarded one of the more unusual kinds of tuberculosis [5].

Some strains of TB have developed resistance to standard antibiotics, making treatment more difficult and requiring longer courses of more potent medications [6-8]. When a healthy person comes into contact with an infected person's cough, spit, or sneeze, the bacteria may enter their body [9, 10]. Even tiny numbers of these bacteria inhaled by a healthy person can cause TB. Despite advances in diagnostic tools and treatment regimens, the disease continues to challenge healthcare systems, particularly in resource-limited settings, due to its diverse clinical manifestations and emerging drug-resistant strains. TB can be treated using First-line drugs like isoniazid, rifampicin, pyrazinamide, ethambutol, *etc.* Multidrug-resistant TB (MDR-TB) and extensively drug-resistant TB (XDR-TB) are the different drug-resistance conditions. With the aid of bioinformatics databases and services, drug targets can be identified, and lead compounds can be analyzed [11]. There are numerous tools, resources, and bioinformatics-based approaches associated with *M. tuberculosis* drug discovery [12].

TB can be diagnosed using different techniques, namely microbiological (smear microscopy, culture), molecular (PCR), radiological (chest X-ray), and immunological (IGRA, Mantoux test). There are many challenges in diagnosing TB in different populations. Current diagnostic approaches, such as sputum microscopy, molecular tests, and chest X-rays (CXR), are frequently constrained by high expenses, time constraints, or the requirement for skilled staff. However, it mostly depends on professional interpretation, which can be time-consuming and subjective. Chest radiographs are one of these widely accessible and reasonably priced TB diagnosis methods. These constraints highlight the necessity for automated, dependable, and scalable diagnostic tools.

An innovative machine-learning framework for the automated classification of pulmonary tuberculosis using CXR pictures is presented in this manuscript. To improve classification accuracy, the suggested method makes use of three different feature extraction techniques: edge, shape, and Gray Level Difference Statistics (GLDS). Our framework stresses the integration of hybrid features (edge + shape + GLDS) with conventional machine-learning classifiers like Support Vector Machines (SVMs), k-nearest Neighbours (kNN), Decision Tree (DT), Logistic Regression, Artificial Neural Networks (ANN), in contrast to previous studies that frequently rely on single-feature approaches or intricate, deep learning models requiring significant computational resources. The main contribution of this work is the establishment of a hybrid feature set that improves the depiction of disease characteristics in chest CXR images by combining edge, shape, and GLDS features. In order to determine the best configuration for TB classification, the performance of the framework was optimized by comparing several classifiers under two distinct dataset splits (70:30 and 80:20). The robustness and generalizability of the proposed approach are also confirmed by testing it on different datasets and comparing it to cutting-edge techniques. Additionally, the study demonstrates the possibility for deployment in low-resource situations by demonstrating the resource efficiency of using typical machine-learning models on both pre-processed and non-pre-processed images. This study underscores the potential of integrating advanced feature extraction techniques with machine learning to aid in early and accurate TB diagnosis. Particularly in high-burden areas, such a strategy might greatly increase radiologists' skills, provide lower diagnostic errors and enhancing patient outcomes.

## 2 LITERATURE REVIEW

With the aid of bioinformatics databases and services, drug targets can be identified, and lead compounds can be analyzed. There are numerous tools, resources, and bioinformatics-based approaches associated with *M. tuberculosis* drug discovery. Early detection can help fight and cure tuberculosis, for which new molecular diagnostic techniques, histological analysis of biopsy samples, and chest computed tomography (CT) can all help with the diagnosis. The most frequently used techniques for TB detection are chest X-rays and sputum smear microscopy. X-ray technique is one of the most efficient imaging methods for identifying fractures, foreign objects, lung and bone diseases, and even for molecular drug discovery.

Duong *et al.* [13] sought to create an intricate framework for the early diagnosis of TB using CXR images by combining cutting-edge machine learning and computer vision techniques. As the main classification methods, the study used three advanced deep neural networks (DNNs): a modified version of the original Vision Transformer, a modified EfficientNet, and a modified Hybrid EfficientNet with Vision Transformer. A large dataset, created by combining several publicly available datasets, was used for assessment and partitioned into training (80%), validation (10%), and testing (10%) sets. The results highlighted the superior prediction performance achieved through Vision Transformer and transfer learning. The ViT_Base_EfficientNet_B1_224 model achieved the highest accuracy of 97.72% with an AUC of 100%, while EfficientNet-B1, trained using Noisy Student weights, ranked second with an accuracy of 97.61%. Ye *et al.* [14] sought to distinguish *M. tuberculosis* inhibitors from non-inhibitors by developing classification models utilizing four machine learning (ML) techniques. To describe the bioactivity of molecules, the system uses six different kinds of molecular descriptors and fingerprints—RDKit descriptors, AtomPairs fingerprints, Morgan fingerprints, MACCS Keys, RDKit fingerprints, and PubChem fingerprints. A total of 24 classification models were developed using SVM, XGBoost, Random Forest (RF), and Deep Neural Network (DNN) algorithms, and two consensus tactics were applied to combine the predictions from multiple models. The best-performing model was the XGBoost model with Morgan and RDKit fingerprints, achieving an AUC of 0.832. Additionally, the consensus model, which combined the predictions of XGBoost, RF, and DNN, yielded the highest AUC scores of 0.842 for the 10-fold cross-validated training set and 0.942 for the external test set. Another research [5] presented an optimized machine-learning approach for extracting texture features from TB CT scan images and determining the hyperparameters of classifiers. Their methodology consisted of four stages: preprocessing input data, feature extraction, feature selection, and SVM hyperparameter tuning. Utilizing the publicly available ImageCLEF 2020 dataset, the study employed a genetic algorithm (GA) to select optimal features, which were then fed into an SVM classifier. Results demonstrated that the improved SVM classifier outperformed previous classification algorithms in TB classification accuracy. The study emphasized the critical impact of high-quality feature extraction on image classification efficiency, with the SVM classifier's mean accuracy peaking when paired with GA-optimized parameter choices. The authors of a study [15] suggested an advanced automated approach for TB detection based on deep learning (DL) models. Their methodology involved using CXR images, where relevant areas were extracted through the U-Net model, and the segmented images were subsequently processed by DL models for feature extraction. The study utilized three publicly available CXR datasets and employed eight distinct CNN models to compare experimental and classification results. The findings demonstrated that segmented lung CXR images combined with ensemble learning achieved superior outcomes compared to unsegmented images. Key metrics achieved using the U-Net model included an intersection over union (IoU) score of 93.10, a dice coefficient score of 96.50, and a segmentation accuracy of 98.58%. Additionally, the proposed stacked ensemble algorithm excelled in sensitivity (98.89%), accuracy (98.38%), and specificity (98.70%). In order to develop an effective technique for TB identification, the authors of [16] employed stochastic learning with CXR pictures to build an ANN model with random modifications. Their methodology incorporated diverse deep geometric contexts, including size, shape, cavitation, and density, to assess varying degrees of TB complexity. The proposed approach demonstrated high effectiveness, achieving an accuracy of 98.45%, specificity of 98.01%, sensitivity of 96.12%, and an F-Score of 95.88%. Three levels of a methodology were proposed in a study: pre-processing, feature extraction, and training & classification [17]. Feature descriptors, including the local binary pattern (LBP) and the histogram of oriented gradients (HOG), were employed to extract relevant features. Various classifiers were applied for classifying COVID-19, TB, and pneumonia, including RF, SVM, XGBoost, DT, Naïve Bayes (NB), AdaBoost, kNN, Logistic Regression (LR), and an Ensemble Model (EM). The combination of three supervised learning algorithms—LR, RF, and XGBoost—achieved the highest classification accuracy of 98%. In contrast, NB, DT, and AdaBoost exhibited subpar performance, with accuracy rates of 78%, 83%, and 84%, respectively. Using a dataset that included numerous clinical and biological characteristics associated with TB [18] assessed the effectiveness of four classification algorithms, *i.e.*, LR, kNN, RF, and Naive Bayes (NB) in identifying TB. The results of the study showed that the Random Forest and kNN algorithms had the highest accuracy of 99.8%, followed by Naive Bayes and Logistic Regression, both of which had 99% accuracy. Sitanggang *et al*. [19] used kNN to assess and categorize patient data pertaining to TB based on a number of clinical and laboratory factors associated with the disease. Using data mining, kNN approach was used to classify tuberculosis with an accuracy of 80%. A classification method for detecting infection in chest X-ray images was put out in another study [20] where a publicly available X-ray database was used and divided into three groups: healthy people, pneumonia patients, and other COVID-19 patients. ML algorithms like SVM, kNN, and RF achieved classification rates of 99.47%, 98.70%, and 98.47%, respectively. Further, one study [21] used a unique, model-less segmentation technique to improve TB identification using chest X-ray images. The study produced encouraging results by using a variety of classifiers, including CNN, SVMs, and kNN, in conjunction with statistical, geometric, and Hog descriptive data extracted from lung images. CNN showed comparable results with an accuracy of 89.58%, while the greatest accuracy of 91.88% was achieved using linear regression and self-extracted features.

The process of automatically identifying TB in chest radiographs by combining textural, focal, and shape abnormalities analysis is described in [22]. The authors explained a novel multiple-instance learning-based method for computer-aided TB identification on CXRs [23]. Feng *et al*. describe how a CT-based deep learning nomogram can be used to distinguish between lung adenocarcinomas & TB granulomas [24]. Primary Renal TB was described by authors in [25] as a giant cyst near the kidney's lower pole. The explanation of [26] includes a transposon insertion mutant of *M. fortuitum* that is complemented by Rv3291c of *M. tuberculosis* and diminished virulence and persistence in a mouse infection model. It was linked to HIV infection by authors in [27, 28]. Primary oral TB was proposed as a sign of HIV infection.

### 2.1 Problem Formulation

A major cause of morbidity and mortality worldwide, tuberculosis (TB) is especially prevalent in areas with insufficient resources [29]. According to the WHO Global TB Report 2024, 10.8 million new cases of tuberculosis were diagnosed in 2023, up from 10.6 million in 2022. The sustained increase is a reflection of the lingering consequences of TB care interruptions during the most severe COVID-19 epidemic years. TB is still the most common infectious disease that kills people worldwide, even more so than COVID-19. Further complicating matters for healthcare systems is the rise of drug-resistant strains, including multidrug-resistant TB (MDR-TB) and extensively drug-resistant TB (XDR-TB). Existing diagnostic procedures, including molecular testing like PCR, sputum microscopy, and imaging techniques like chest X-rays (CXR), are useful, but they have limitations. Particularly in some groups, sputum-based testing may be insensitive and time-consuming. Even while CXR is readily accessible and reasonably priced, its interpretation by qualified radiologists can be subjective and error-prone, especially in environments with limited resources [29]. The healthcare industry is exploring artificial intelligence (AI) and machine learning (ML) technologies in response to the need for automated, precise, and scalable diagnostic solutions. Traditional machine-learning methods, when combined with engineered features, offer a practical solution to the limitations of chest radiographs [30-33].

In order to increase the accuracy of TB classification using CXR images, authors have used different machine learning techniques on pre-processed and non-processed images, and the results are compared based on attained accuracy. A unique framework that makes use of hybrid feature extraction techniques (edge, shape, and GLDS) has been suggested because of its emphasis on resource efficiency; the framework is well-suited for environments with limited resources. By enabling early and accurate disease detection, our initiative seeks to close the gap between automated TB diagnosis accuracy and accessibility, supporting the WHO's End TB Strategy. The creation of a thorough framework for the automated classification of pulmonary TB via chest X-ray images that incorporates cutting-edge feature extraction methods and machine-learning models is what makes this study novel. The paper presents a hybrid feature set that combines edge, shape, and GLDS features. The framework's assessment across several machine-learning classifiers (SVM, kNN, ANN, *etc.*) and dataset splits (70:30 and 80:20) sets it apart further and enables an extensive performance study.

This paper is organized as follows: section 3 explains the methodology used in detecting TB using different machine learning techniques considering different features and splitting of data, section 4 discusses the results obtained after considering different techniques, and section explains the concluding remarks followed by future work.

## 3 MATERIALS AND METHODOLOGY

Initial diagnosis of TB results in an earlier beginning of drug therapy, a shorter time of infectiousness, and better patient outcomes. Doctors will discover a patient's cough during a physical examination if they have TB. This kind of cough results in phlegm production. Other symptoms include tiredness, loss of appetite, and eventually weight loss [34, 35]. A defining feature of active TB is the development of cavitary lesions in the lungs. The bacteria usually attack the lungs, but they can also damage other parts of the body. TB spreads through the air when a person with TB of the lungs or throat coughs, sneezes, or talks [36, 37]. TB disease symptoms are often initially mistaken for a smoker’s cough, allergies, or chronic bronchitis from a lingering cold or flu infection. TB infection most often affects the lungs but can cause problems in other parts of the body. The different types of TB infection are shown in Fig. (**1**).

TB can be latent or active after infection [38, 39]. When TB is working in an active state, the bacteria invade the body and spread the infection. During the latent infection, the disease does not spread and remains dormant in the body for an extended period, restraining the disease from occurring. Although pulmonary TB primarily affects the lungs, extrapulmonary TB may invade other places of the body [40, 41]. TB is a disease that has a significant fatality rate if left untreated. Pulmonary TB occurs when *M. tuberculosis* primarily attacks the lungs [42, 43]. However, it can spread from there to other organs. Pulmonary TB is curable with an early diagnosis and antibiotic treatment. If left untreated, the disease can cause life-threatening complications like permanent lung damage [44, 45]. When these bacteria enter the lungs, they are usually walled off into harmless capsules (granulomas) in the lung, causing infection but not disease. Some species are also associated with animals. While many *mycobacteria* are harmless saprophytes, some species are pathogenic to humans and animals, causing a range of diseases. The most well-known pathogenic *mycobacteria* include:

i. *M. tuberculosis*: The contributory agent of TB, one of the most significant infectious diseases worldwide.

ii. *M. leprae*: The causative agent of leprosy (Hansen's disease), which affects the skin and nerves.

iii. *M. avium complex* (MAC): Includes several closely related species, such as *M. avium* and *M. intracellulare*, which can cause opportunistic infections, particularly in immunocompromised individuals, like HIV/AIDS.

iv. *M. ulcerans*: The causative agent of Buruli ulcer, a skin disease prevalent in certain regions of Africa, Australia, and other parts of the world.

v. *M. bovis*: Causes TB in cattle and can also infect humans, primarily through consumption of contaminated dairy products or direct contact with infected animals.

vi. *M. abscessus*: A rapidly growing mycobacterium associated with skin and soft tissue infections, pulmonary infections, and other systemic infections, particularly in immune-compromised individuals.

The methodology employed in this research article is depicted in Fig. (**2**).

The authors considered different databases of CXR images for healthy persons and TB-positive patients. A team of researchers from Qatar University, Doha, Qatar, and the University of Dhaka, Bangladesh, along with their collaborators from Malaysia in collaboration with medical doctors from Hamad Medical Corporation and Bangladesh, have created a database of CXR images for TB-positive cases along with healthy images [45]. In our current release, there are 700 TB images (306 CXR images from the BELARUS TB portal program dataset [46], 394 CXR images from NLM [47], and 3500 healthy images (406 CXR images collected from NLM [47] and 3094 CXR images are collected from RSNA [48]). CXRs are inexpensive diagnostic instruments, to screen for TB. It has a lot of information that is helpful in the easy identification of lung conditions. However, the interpretation of a chest radiograph takes knowledge and skill. It is crucial to treat TB promptly to lower the death rate and stop the infection from spreading. The method of detecting TB automatically from CXRs would be very intriguing to help overworked medical professionals screen an immense population. This is possible by segmenting, examining, and filtering by a computer equipped with image processing features to enhance the efficacy of the diagnosis process by enabling a Neural Network (NN) to identify the existence of TB [49, 50]. There are four categories of image pre-processing methods, namely:

i. Pixel brightness Transformation

ii. Geometric Transformation

iii. Local neighborhood of the processed pixel.

iv. Image Restoration

Each image is subjected to a variety of image processing steps, such as binarization, resizing, thresholding, normalization, *etc.*, before undergoing image feature extraction. Shape, color, texture, and object arrangement provide the foundation of the majority of retrieving image systems [51, 52]. A vital process within the processing of images is feature extraction. It involves finding and displaying distinctive patterns in an image. In feature extraction, unprocessed visual data is converted into numerical features, allowing for processing without losing any important information. These properties are essential for several subsequent processes, including image matching, object detection, and classification [53-55]. The features like edge features, GLDS, and shape features were evaluated, and later, the hybrids of the three were used in this manuscript.

i. *Edge Detection*: In the fundamental domains of image processing, such as feature extraction and detection, edge detection is a crucial technique. An image contains a variety of data, including the object's size, orientation, shape, and color. An image is the input for image processing, and the output can be an image or a set of parameters or characteristics. Changes in light, color, shade, and texture can produce an edge, and these variations can be employed to adjust a digital image's surface qualities, depth, size, and orientation. Therefore, it is necessary to identify the edges that the object involves to determine its shape. The process of identifying edges and their locations is known as *edge detection*. Edges are produced by sudden, sharp changes in an image's brightness or color intensity. Mean, and variance are the features evaluated using this technique. Algorithm 1 explains the process of evaluating edge features.

Algorithm 1: Process of evaluating edge features

Input Images

Output edge features

Start

Step 1 Read the images.

Step 2 Convert RGB images to gray images.

Step 3 Evaluation of horizontal edge image from Sobel mask.

Step 4 Computation of probability distribution of grey levels.

Step 5 Computation of mask to select a region of interest if mask =1 (representing the inside region), and 0 elsewhere.

Step 6 Initialize grey level count vector.

Step 7 Add 1 to all pixels and mask out unwanted regions, so that a pixel value of 0 occurs only outside the region of interest, and add 1 again to give an index into the count vector (loops to work through the image to count the pixels of each grey level).

Step 8 Discard the first element of P, which counts pixels outside the area of interest.

Step 9 Normalize P into a probability distribution.

Step 10 Calculate edge features (mean, variance).

Step 11 Write edge features in Excel.

End

Though there are multiple approaches to edge detection, they are, nevertheless, can be divided into two types. Gradient-based edge detection and Laplacian-based edge detection. Using the gradient-based method, edges are identified by locating the maximum and lowest in the image's first-order derivative. The edge strength is determined by calculating the gradient magnitude. The local directional maxima qualities of gradient magnitude aid in the computation of the local edge orientation, which is in the gradient direction. The second-order derivative equation in the Laplacian-based technique is computed using an image that includes a zero crossing. The Laplacian strategy looks for zero crossings in the image's second derivative to find edges. Finding the zero crossing of a nonlinear differential expression is typically how edges are located. A ramp's one-dimensional shape represents an edge, and its location can be highlighted by calculating the derivative of the image.

ii. *Shape Features*: Shape is a fundamental characteristic that is employed to recognize and classify the content of a picture. Shape features are a common visual cue used by people to compare and contrast things. They are either based on shape boundary information or boundary along with inner information, which is the primary basis for shape depiction. Shape features can be split into two categories namely contour-based (CB) and region-based (RB). Both techniques are further classified into two categories, *i.e.*, global methods and structural methods based on how they use the contour or region information; whether they use it all at once or divide it up for representation and description. The contour features are assumed to be the primary means by which humans distinguish shapes; CB methods are more often used than RB methods. CB approaches are commonly employed in shape applications where the contour of the shape is more significant than its inside content. RB methods are more reliable than CB methods as they make use of all the given shape information. Retrieval accuracy is increased with RB approaches because RB approaches utilize all of the shape's accessible information. They can overcome the issue of shape defection, which is not handled well by CB methods. Bounding Box, Area, Centroid, Perimeter, Diameter, Major Axis Length, Minor Axis Length, Eccentricity, Orientation, Convex Area, and Extent were evaluated as Shape features. Algorithm 2 explains the process of evaluating shape features.

Algorithm 2: Process of evaluating shape features

Input Images

Output shape features

Start

Step 1 Read the images

Step 2 Convert RGB images to Gray images

Step 3 Evaluation of horizontal edge image from Sobel mask

Step 4 Computation of probability distribution of grey levels

Step 5 Computation of mask to select a region of interest mask=1 (inside the region), and 0 elsewhere

Step 6 Initialize grey level count vector

Step 7 Add 1 to all pixels and mask out the unwanted region, so that a pixel value of 0 occurs only outside the region of interest, and add 1 again to give the index into the count vector

Step 8 Discard the first element of P which counts pixels outside the area of interest

Step 9 Normalize P into a probability distribution

Step 10 Convert to Binary and fill the holes

Step 11 Label the image an Object Number

Step 12 Find the Bounding Box, Area, Perimeter, diameter, and roundness

Step 13 Calculation with 'region props'(For verification Purpose);

Step 14 Measure the solidity of all the blobs, sort in order of decreasing solidity, and get the solidity of the most solid blob

Step 15 Get the label of the most solid blob (Major Axis Length, Minor Axis Length, Eccentricity, Orientation; Convex Area; Extent)

Step 16 Evaluate shape Features Values

Step 17 Write edge features in Excel.

End

iii. *Textural Features:* The structural patterns of materials like sand, fabric, grain, grass, and wood exhibit texture. Repetition of the fundamental texture components, or texels, is referred to as texture in general. A texel is made up of several pixels that may be arranged randomly or periodically. Artificial textures are frequently periodic, while natural textures are typically random. The human visual system uses texture to identify and comprehend images. The homogeneity quality of texture essentially explains the patterns seen in images. Color is typically a pixel attribute, whereas texture can only be quantified from a collection of pixels. Many methods have been put forth for obtaining texture features. They can be broadly divided into two categories: spectral & spatial texture feature extraction methods depending on the field within which the texture feature is extracted. An image is processed into the frequency domain using the spectral texture feature extraction approach, and features are subsequently calculated from the altered image. The spatial texture feature extraction approach helps in retrieving texture characteristics by allowing the calculation of the pixel statistics or by locating the local pixel structures in the initial image domain. The primary goal of the texture classification section in texture analysis is to offer descriptors for classifying textural images. Feature extraction approaches, like statistical methods, can be used to extract some aspects that are not readily recognizable. GLDS is part of the statistical method of texture classification. Absolute differences between gray-level sets serve as the basis for gray-level differences. A vector of 256 elements represents these variations. They are calculated by counting the instances in which the difference is 0, 1,..255 by determining the absolute differences of all feasible pairs of gray levels spaced at a distance apart at an angle ‘Ѳ’. To normalize the difference statistics, divide each vector element by the total number of potential pixel pairs. A histogram of the differences in gray levels is applied to gather various texture characteristics like mean, entropy, contrast, homogeneity, and energy. Algorithm 3 explains the process of evaluating GLDS features.

Algorithm 3: Process of evaluating GLDS features

Input Images

Output GLDS features

Start

Step 1 Read the images

Step 2 Convert RGB images to Gray images.

Step 3 Calculate the probability distribution of grey level differences for displacement distance d at four different angles (0̊, 45̊, 90̊ and 135̊).

Step 4 Compute mask to select a region of interest where mask = 1 (represents inside the region), and 0 elsewhere

Step 5 Initialize grey level difference count vector

Step 6 calculate grey level differences + 1 so that grey level difference = 0 can only arise outside the region of interest after multiplication by masks at any four different angles (0̊, 45̊, 90̊ and 135̊).

Step 7 Normalize grey level difference histogram for all angle

Step 8 Calculate GLDS features

Step 9 Write edge features in Excel.

End

Features such as edge, shape, and GLDS are chosen in the work as they provide indispensable details regarding tuberculosis identification in chest X-ray (CXR) images. In these radiographs, TB frequently appears as aberrant forms, textures, and structural alterations such as cavitation, nodules, and fibrosis. Edge and shape features capture these structural irregularities, which offer crucial diagnostic data. Edge detection is the process of identifying the lines that distinguish the appearance of an image from other regions or objects inside a digital image. To help differentiate important locations like lung cavities or infiltrates, edges draw attention to the borders of lesions or aberrant regions. It facilitates the extraction of beneficial characteristics for pattern recognition from an image by utilizing factors such as blur, noise, objects, illumination, and intensity. Edge detection strives to assess the shape features and the reflectance of the image. Shape characteristics, including contours or geometric features, help detect abnormalities brought on by tuberculosis, like modifications to the size or structure of the lungs [56, 57]. They improve diagnostic accuracy by aiding in the localization of diseased regions, such as lesion forms and lung boundaries. Textural characteristics like granularity and smoothness, as well as local intensity changes, are captured by GLDS. Subtle abnormalities in the lungs can be identified effectively by GLDS, which could otherwise be left unrecognized [58, 59].

A specialist area of computer science called AI researches and creates smart devices [60-62]. The term originated in the 1950s in the US. It involves computers, more particularly, machines, emulating human cognitive capacities. Because AI can evaluate vast volumes of data from various sources, it can potentially improve the healthcare industry. According to Arthur Samuel in 1959, ML is a subtype of AI that enables computers to pick up information from past data or occurrences without the need for specialized programming. Making it possible for machines to extract meaning from data and provide accurate outcomes is the primary goal of ML [63, 64]. ML requires the formulation of algorithms for accurate outcome variable prediction. Machine learning comprises supervised, semi-supervised, unsupervised, reinforcement learning, deep learning, feature engineering, model evaluation and validation, and hyperparameter tuning.

The supervised form of machine learning involves using sample input-output pairs to train a function that relates an input to an output. It uses classification and regression methods for result predictions [65, 66]. Supervised learning is implemented when certain objectives are determined to be achieved from a particular set of inputs. Popular supervised learning techniques include DT, RF, LR, and SVM. Unsupervised learning is a data-driven method that examines unlabeled datasets without requiring human intervention. Few features are obtained from the data by the unsupervised learning algorithms [67-69]. Its primary applications are in feature reduction and clustering. This is frequently used to organize findings, extract generative features, and uncover significant trends and structures. As semi-supervised learning uses both labeled and unlabeled data, it can be thought of as a combination of the supervised and unsupervised approaches. Some uses for semi-supervised learning are the classification of text, automated translation, identifying fraudulent activity, and data labeling. One kind of machine learning technique called reinforcement learning allows computers and software programs to automatically assess the best behavior in a certain setting or context to increase efficiency [70-72]. Through reinforcement learning, the optimal effectiveness of a given environment is ascertained in an “environment-driven approach.” Deep learning offers a computational architecture to learn from data by merging many processing levels, including input, hidden, and output layers. It performs better in several scenarios, especially when learning from big datasets. Feature engineering involves selecting, adjusting, or creating new features from raw data to improve model performance [73, 74]. A detailed analysis is required to ascertain the effectiveness and generalization potential of a machine learning model. Model evaluation techniques include holdout validation, cross-validation, and measures like precision, recall, accuracy, ROC-AUC curve, and F1-score. SVMs are conventional machine-learning models that perform outstandingly in binary classification tasks by identifying the best hyperplane for class separation. When features like shape, edge, or texture are appropriately extracted, as they were in this work, they can achieve high accuracy. ANN, DT, and kNN are examples of machine learning models that use engineered features to provide interpretable outcomes. In situations where computational capacity is restricted, this study emphasizes how scalable solutions for TB detection can be achieved by combining traditional machine learning techniques with feature extraction methods. SVMs and ML approaches are useful for developing readily available, interpretable, and adaptable solutions in regions with limited resources.

## 4 RESULTS AND DISCUSSION

TB detection using AI primarily revolves around analyzing medical images, such as CXRs or CT scans, and sometimes using AI algorithms on clinical data. AI holds significant promise in improving TB detection through advanced image analysis and integration with clinical data, ultimately contributing to early diagnosis and better management of the disease. However, it's crucial to validate AI algorithms rigorously to ensure they are deployed ethically and responsibly in accordance with clinical practice. The authors used the publicly available dataset of CXR images collected from NLM [47-50]. Figs. (**3** and **4**) show the different processing techniques for normal and infected images, respectively. The pre-processing phase began when images were resized to 224 × 224 × 3 and then transformed into an array to hold the values. The values of an array were all rescaled by 255. In this manuscript, authors used average, weighted average, mean filter, gaussian filter, and different transformations as processing techniques. By applying a blur filter, high-frequency noise components are smoothed out, resulting in a reduction of noise.

Figs. (**3** and **4**) show the results of pre-processing considering average filter with different kernel sizes, weighted average filter, mean filter, gaussian filter with different filter sizes, line and edge detectors, and different transformations like contrast stretching, adjust, and log transforms. The authors contemplated detecting the precise area in the image wherein the disease condition is present by utilizing bounding boxes. The differential diagnosis of TB is a challenge, even for experienced radiologists. Therefore, a Computer-Aided Design (CAD) system for the classification of different TB is highly desirable. In light of this fact, a CAD system design is proposed to evaluate the performance of different classifiers. Logistic regression, kNN, Decision tree, ANN, and SVM with different kernels (linear, RBF, and poly) are the classification techniques utilized. The dataset was divided into sets for training and testing, considering 70: 30 and 80: 20. Utilizing the training set, the processed images were trained, and then results were assessed on the testing set, and the accuracy was evaluated.

Tables **1**-**4** list the various experiments conducted for this article.

### 4.1 An Experiment Carried Out for Two-class Classification of Tuberculosis using Edge Parameters

This experiment assesses the classification performance of two-class classification of TB using Edge parameters considering without pre-processing and with pre-processing images. Table **5** displays the experiment's outcomes.

The maximum accuracy attained using SVM with linear kernel was 83%, and the minimum accuracy using logistic regression was 79.7%, considering pre-processed images for edge features.

### 4.2 An Experiment Carried Out for Two-class Classification of Tuberculosis using GLDS Parameters

This experiment assesses the classification performance of two-class classification of TB using GLDS parameters considering without pre-processing and with pre-processing images. Table **6** displays the experiment's outcomes.

The maximum accuracy attained using SVM with linear kernel was 85%, and the minimum accuracy using logistic regression was 79.5%, considering pre-processed images for GLDS features.

### 4.3 An Experiment Carried Out for Two-class Classification of Tuberculosis using Shape Parameters

This experiment assesses the classification performance of two-class classification of TB using shape parameters considering without pre-processing and with pre-processing images. Table **7** displays the experiment's outcomes.

The maximum accuracy attained using SVM with linear kernel was 90%, and the minimum accuracy using logistic regression was 83.75%, considering pre-processed images for the shape feature.

### 4.4 An Experiment Carried Out for Two-class Classification of Tuberculosis using Shape + edge + GLDS Parameters

This experiment assesses the classification performance of two-class classification of TB using shape + edge + GLDS parameters considering without pre-processing and with pre-processing images. Table **8** displays the experiment's outcomes.

The best accuracy was obtained at 93.5% using an SVM linear kernel with hybrid parameters. The linear kernel works great when having a lot of features because then data is already linearly separable and an SVM will find the best-separating hyperplane. Linear kernels work well for very sparse data like text. 92.6% accuracy was attained using the SVM RBF kernel with hybrid parameters. The RBF kernel has a hyperparameter called the gamma parameter, which controls the shape of the decision boundary and the amount of smoothing in the model. If the gamma parameter is too high, the model may overfit the training data and perform poorly on the test data. If the gamma parameter is set too low, the model may underfit the data and not capture the underlying patterns. Percentage improvement with all the techniques is shown in Table **9** considering hybrid features as base.

Edge features show more values of percentage improvement. Comparison with other work is discussed in the next section. Edge features display percentage improvement in accuracy values. Classifier performance is greatly impacted by the pre-processing methods, which include scaling photos, normalizing data, and using filters like Gaussian blur to improve data quality and lower noise. Consistent results from validation testing on other datasets, such as the Shenzhen dataset, support the relevance of the suggested methods.

## 5 COMPARISON AND VALIDATION OF RESULTS

The designed model using SVM, decision tree, kNN, ANN, and logistic regression was compared using other state-of-the-art techniques, other datasets, different feature extraction techniques, and different splitting of data.

### 5.1 Comparison with other Datasets

The proposed model was validated with other datasets [60], and the results are presented in Table **10**.

It was observed that the dataset from Qatar resulted in 93.5% accuracy, and from Shenzen, it resulted in 92.4%. In the next section, a comparison with different feature extraction techniques was done.

### 5.2 Comparison with Different Feature Extraction Techniques

The comparison of accuracies for pre-processed images with different feature extraction techniques was done and is shown in Fig (**5**).

Fig. (**5**) compares the accuracy of unlike FE techniques and it was observed that maximum accuracy was attained using hybrid features.

### 5.3 Comparison with Different Values of Splitting of Dataset

Authors consider different values of dividing the dataset into training and testing data *i.e.*, 70:30 and 80:20. The results are shown in Fig. (**6**).

It was observed that maximum accuracy was attained using 70:30 for the SVM linear kernel considering hybrid parameters. The best accuracy considering all the combinations is tabulated in Table **11**.

The study uses several classifiers (such as SVM, Decision Tree, kNN, ANN, and Logistic Regression) based on a number of parameters (edge, shape, GLDS, and hybrid features), resulting in performance differences among these classifiers in particular situations. SVM with a linear kernel, for example, demonstrates its durability and adaptability for linearly separable data by achieving the best accuracy of 93.5% when employing hybrid features (edge + shape + GLDS) and a 70:30 train-test split. In contrast, kNN demonstrates its dependability with discrete, well-pre-processed data by achieving 91% accuracy with hybrid features. Hybrid features are especially effective since they combine complementing data to outperform single-feature approaches. In areas with limited healthcare resources, AI-powered diagnostic tools can facilitate remote diagnosis and consultation. This can potentially extend the reach of healthcare services and improve access to timely TB detection. AI is used in TB research for drug discovery, predicting treatment outcomes, and understanding disease progression. AI models can analyze vast amounts of genomic, clinical, and epidemiological data to uncover insights that aid in combating TB. These algorithms can complement human radiologists by providing a second opinion or highlighting subtle features that may be missed during manual interpretation. This can improve diagnostic accuracy and reduce the rate of false negatives or false positives.

## CONCLUSION

For prompt treatment and a decrease in mortality rates, early identification of tuberculosis is essential. This study offers a solid foundation for the automated categorization of tuberculosis from CXR images that makes use of both conventional machine-learning models and sophisticated feature extraction approaches. A comprehensive representation of disease characteristics has been suggested thanks to its novel hybrid feature set that combines edge, shape, and GLDS features. A thorough performance study is also made possible by the framework's examination across a variety of machine-learning classifiers (SVM, kNN, ANN, LR, DT) and dataset splits (70:30, 80:20), demonstrating its adaptability and resilience. With a 70:30 dataset split, the hybrid feature set that combined edge, shape, and GLDS characteristics with the SVM classifier performed the best, attaining 93.5% accuracy. 0.64% improvement was observed when considering the 70:30 dataset in place of 80:20 with hybrid features. The outcomes confirm that this method, which uses both pre-processed and non-pre-processed photos for model training and testing, is feasible, particularly in low-resource environments. The proposed method offers a comprehensive representation of disease characteristics thanks to its novel hybrid feature set that combines edge, shape, and GLDS features. This method offers a resource-friendly, scalable, and effective automated TB detection option, which is especially helpful in environments with limited resources. Additionally, this study can be used as a basis for directing resources in the early management of TB patients.

Future research will concentrate on adding more varied cases to the dataset, using metaheuristic approaches for feature selection, model fusion techniques, and creating an intuitive diagnostic tool for real-time tuberculosis identification.

## Figures and Tables

**Fig. (1):**
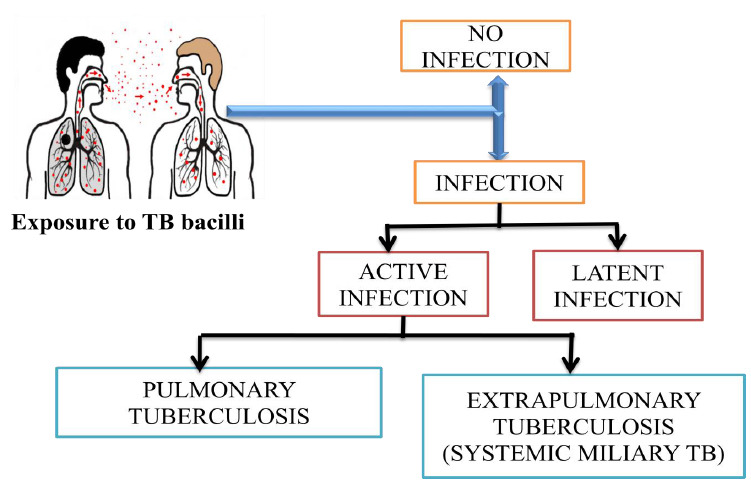
Types of TB infection.

**Fig. (2):**
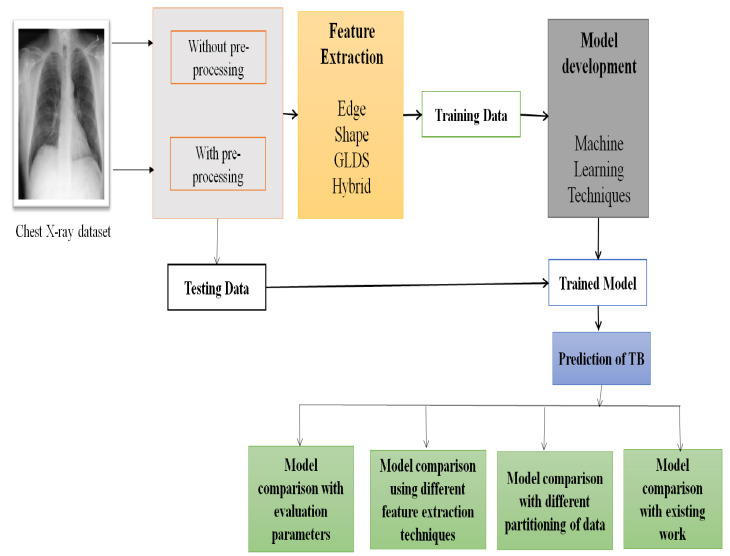
Proposed methodology for two-class classification of pulmonary tuberculosis using machine learning.

**Fig. (3):**
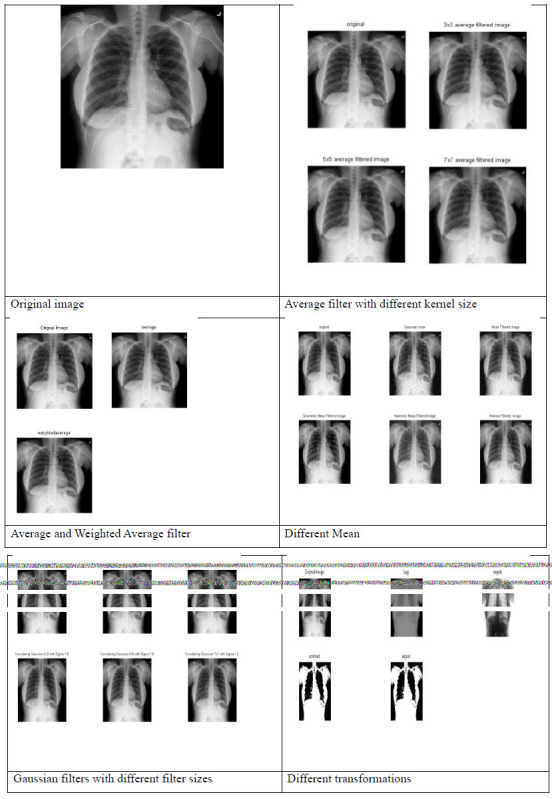
Different processing techniques for normal image.

**Fig. (4):**
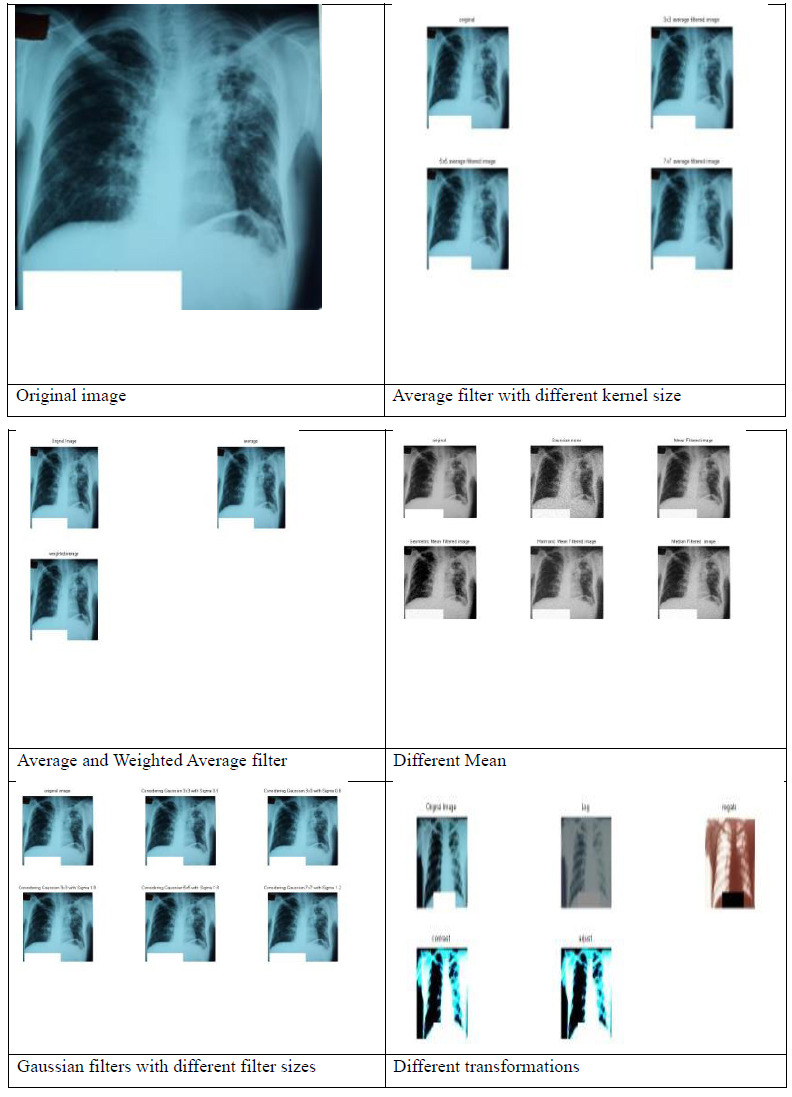
Different processing techniques for TB image.

**Fig. (5):**
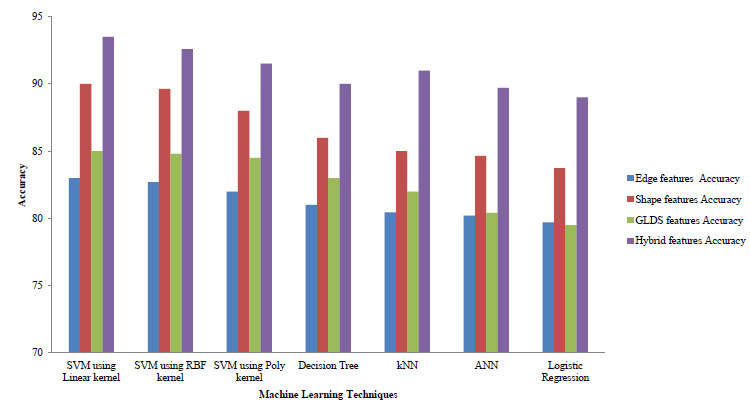
Comparison with different FE techniques.

**Fig. (6):**
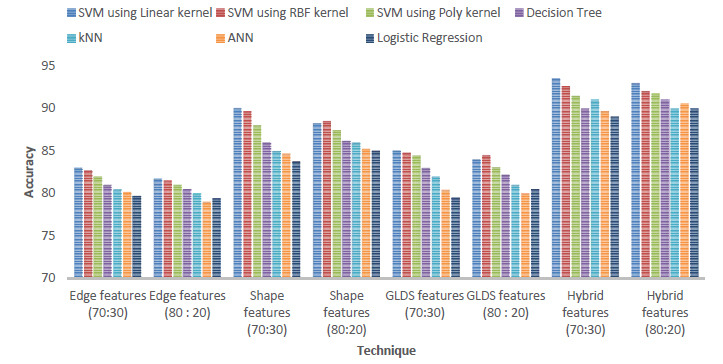
Accuracy values for different testing and training data.

**Table 1:** An explanation of the experiments done to classify tuberculosis using Edge parameters.

Experiment: To attain the classification performance of Edge parameters for two-class classification using Logistic regression, Decision tree, kNN ANN, and SVM classifiers.

**Table 2:** An explanation of the experiments done to classify tuberculosis using GLDS parameters.

Experiment: To obtain the classification performance of GLDS parameters for two-class classification using Logistic regression, Decision tree, kNN ANN, and SVM classifiers.

**Table 3:** An explanation of the experiments done to classify tuberculosis using shape parameters.

Experiment: To obtain the classification performance of shape parameters for two-class classification using Logistic regression, Decision tree, kNN ANN, and SVM classifiers.

**Table 4:** An explanation of the experiments done to classify tuberculosis using shape + edge + GLDS parameters.

Experiment: To obtain the classification performance of shape + edge + GLDS parameters for two-class classification using Logistic regression, Decision tree, kNN ANN, and SVM classifiers.

**Table 5:** Accuracy values considering Edge parameters.

	**Accuracy without Pre-processing**	**Accuracy with Pre-processing**
SVM using linear kernel	79.4	83
SVM using RBF kernel	79.7	82.7
SVM using poly kernel	79	82
Decision tree	78	81
kNN	77.5	80.45
ANN	75	80.2
Logistic regression	75.5	79.7

**Table 6:** Accuracy values considering GLDS parameters.

	**Accuracy Without Pre-processing**	**Accuracy With Pre-processing**
SVM using linear kernel	77	85
SVM using RBF kernel	77.2	84.8
SVM using poly kernel	76.8	84.5
Decision tree	75	83
kNN	74.6	82
ANN	74	80.4
Logistic regression	72..5	79.5

**Table 7:** Accuracy values considering shape parameters.

	**Accuracy without Pre-processing**	**Accuracy with Pre-processing**
SVM using linear kernel	75	90
SVM using RBF kernel	74.9	89.64
SVM using poly kernel	74.7	88
Decision tree	73	86
kNN	72	85
ANN	70.1	84.66
Logistic regression	69.5	83.75

**Table 8:** Accuracy values considering Edge + Shape+ GLDS parameters.

	**Accuracy without Pre-processing**	**Accuracy with Pre-processing**
SVM using linear kernel	82	93.5
SVM using RBF kernel	82.4	92.6
SVM using poly kernel	81.9	91.5
Decision tree	81	90
kNN	82	91
ANN	81.1	89.7
Logistic regression	80	89

**Table 9:** Percentage improvement.

	**% Accuracy** **(Edge features)**	**% Accuracy** **(Shape features)**	**%Accuracy** **(GLDS features)**
SVM using linear kernel	11.230	3.743	9.091
SVM using RBF kernel	11.551	4.128	9.305
SVM using poly kernel	12.299	5.882	9.626
Decision tree	13.369	8.021	11.230
kNN	13.957	9.091	12.299
ANN	14.225	9.455	14.011
Logistic regression	14.759	10.428	14.973

**Table 10:** Comparison with other datasets.

**Classifiers\Refs.**	**Techniques Used**	**Accuracy (%)**
Tuberculosis Chest X-rays (Kaggle) [45, 47]	SVM using a linear kernel with hybrid features (shape + edge + GLDS)	93.5%
Tuberculosis Chest X-rays (Shenzhen) [75]	SVM using a linear kernel with hybrid features (shape + edge + GLDS)	92.4%

**Table 11:** Comparison of optimized values.

	Hybrid Features (70:30)	Hybrid Features (80:20)
SVM using linear kernel	**93.5**	**92.9**
SVM using RBF kernel	92.6	92
SVM using poly kernel	91.5	91.7
Decision tree	90	91
kNN	91	90
ANN	89.7	90.5
Logistic regression	89	90

## Data Availability

The data and supportive information are available within the article.

## LIST OF ABBREVIATIONS

SVM: Support Vector Machine
ML: Machine Learning
TB: Tuberculosis
kNN: k-nearest neighbour
CXR: Chest X-ray
AI: Artificial Intelligence
NTM: *Non-tuberculous Mycobacteria*
MTBC: *Mycobacterium tuberculosis* Complex
WHO: World Health Organization
DL: Deep learning
NB: Naïve Bayes
HOG: Histogram of oriented gradients
LBP: Local binary pattern
IoU: Intersection over union

## References

[1] Rahlwes K.C., Dias B.R.S., Campos P.C., Alvarez-Arguedas S., Shiloh M.U. (2023). Pathogenicity and virulence of *Mycobacterium tuberculosis*..

[2] Self-study modules on tuberculosis transmission and pathogenesis of tuberculosis’.. https://www.cdc.gov/tb/hcp/education/self-study-modules-on-tuberculosis.html.

[3] Kashyap V.K., Gupta R.K., Shrivastava R., Srivastava B.S., Srivastava R., Parai M.K., Singh P., Bera S., Panda G. (2012). *In vivo* activity of thiophene-containing trisubstituted methanes against acute and persistent infection of non-tubercular *Mycobacterium fortuitum* in a murine infection model.. J. Antimicrob. Chemother..

[4] 2024 Global tuberculosis report.. https://www.who.int/teams/global-tuberculosis-programme/tb-reports/global-tuberculosis-report-2024.

[5] Hrizi O., Gasmi K., Ben Ltaifa I., Alshammari H., Karamti H., Krichen M., Ben Ammar L., Mahmood M.A. (2022). Tuberculosis disease diagnosis based on an optimized machine learning model.. J. Healthc. Eng..

[6] Zhang D., Islam M.M., Lu G. (2012). A review on automatic image annotation techniques.. Pattern Recognit..

[7] Yang N.J., Chiu I.M. (2017). Bacterial signaling to the nervous system through toxins and metabolites.. J. Mol. Biol..

[8] Kroner G.M., Wolfe M.B., Freddolino P.L. (2019). Escherichia coli Lrp regulates one-third of the genome *via* direct, cooperative, and indirect routes.. J. Bacteriol..

[9] Zhang Y., Yang J., Bai G. (2018). Regulation of the CRISPR-associated genes by Rv2837c (CnpB) *via* an orn-like activity in tuberculosis complex *mycobacteria*.. J Bacteriol.

[10] Yang J., Zhang L., Qiao W., Luo Y. (2023). *Mycobacterium tuberculosis*: Pathogenesis and therapeutic targets..

[11] Khandelwal A.S.P.K.A.R.S.I. (2019). Bioinformatics database resources.. Biotechnology: Concepts, Methodologies, Tools, and Applications.

[12] Chauhan R.S., Chanumolu S.K., Rout C., Shrivastava R. (2013). Can *mycobacteria*l genomics generate novel targets as speed-breakers against the race for drug resistance.. Curr. Pharm. Des..

[13] Duong L.T., Le N.H., Tran T.B., Ngo V.M., Nguyen P.T. (2021). Detection of tuberculosis from chest X-ray images: Boosting the performance with vision transformer and transfer learning.. Expert Syst. Appl..

[14] Ye Q., Chai X., Jiang D., Yang L., Shen C., Zhang X., Li D., Cao D., Hou T. (2021). Identification of active molecules against *Mycobacterium tuberculosis* through machine learning.. Brief. Bioinform..

[15] Kotei E., Thirunavukarasu R. (2022). Ensemble technique coupled with deep transfer learning framework for automatic detection of tuberculosis from chest x-ray radiographs.. Healthcare.

[16] Urooj S., Suchitra S., Krishnasamy L., Sharma N., Pathak N. (2022). Stochastic learning-based artificial neural network model for an automatic tuberculosis detection system using chest x-ray images.. IEEE Access.

[17] Amin S.U., Taj S., Hussain A., Seo S. (2024). An automated chest X-ray analysis for COVID-19, tuberculosis, and pneumonia employing ensemble learning approach.. Biomed. Signal Process. Control.

[18] Priyono E. (2024). Prediction of tuberculosis patients with machine learning algorithms.. IPI: Sci. J. Informatics Res. & Learn..

[19] Sitanggang D., Simangunsong L., Sundah G.F., Hutahaean R., Indren I. (2024). Application of data mining for tuberculosis disease classification using K-nearest neighbor.. Sisfokom J..

[20] Albanhawy M.M., Khalil A., Moustafa H. (2024). Improving life-threatening lung diseases classification using hybrid smote-enn with assorted machine learning classifiers.. Int. J. of Telecommun..

[21] Ali R. (2024). Diagnosis of pulmonary tuberculosis by posterior-anterior lung X-ray.. J. Comput. & Biomed. Informatics.

[22] Hogeweg L., Sánchez C.I., Maduskar P., Philipsen R., Story A., Dawson R., Theron G., Dheda K., Peters-Bax L., van Ginneken B. (2015). Automatic detection of tuberculosis in chest radiographs using a combination of textural, focal, and shape abnormality analysis.. IEEE Trans. Med. Imaging.

[23] Melendez J., van Ginneken B., Maduskar P., Philipsen R.H.H.M., Reither K., Breuninger M., Adetifa I.M.O., Maane R., Ayles H., Sánchez C.I. (2015). A novel multiple-instance learning-based approach to computer-aided detection of tuberculosis on chest X-rays.. IEEE Trans. Med. Imaging.

[24] Feng B., Chen X., Chen Y., Lu S., Liu K., Li K., Liu Z., Hao Y., Li Z., Zhu Z., Yao N., Liang G., Zhang J., Long W., Liu X. (2020). Solitary solid pulmonary nodules: A CT-based deep learning nomogram helps differentiate tuberculosis granulomas from lung adenocarcinomas.. Eur. Radiol..

[25] Singh S., Kumar M., Kumar A., Kumar S., Sankhwar S.N. (2017). Primary renal tuberculosis presented as giant cyst at lower pole of kidney.. Int. J. Life-Sci. Sci. Res..

[26] Parti R.P.S., Shrivastava R., Srivastava S., Subramanian A.R., Roy R., Srivastava B.S., Srivastava R. (2008). A transposon insertion mutant of *Mycobacterium fortuitum* attenuated in virulence and persistence in a murine infection model that is complemented by Rv3291c of *Mycobacterium tuberculosis*.. Microb. Pathog..

[27] Khammissa RAG. (2011). Primary oral tuberculosis as an indicator of HIV Infection.. Pathol. Res. Int..

[28] Srivastava S., Sinha A., Kamala R., Srivastava A. (2011). Primary tuberculosis of the oral cavity.. Indian J. Dent. Res..

[29] Oloko-Oba M., Viriri S. (2022). A systematic review of deep learning techniques for tuberculosis detection from chest radiograph.. Front. Med..

[30] Lee J.H., Park S., Hwang E.J., Goo J.M., Lee W.Y., Lee S., Kim H., Andrews J.R., Park C.M. (2021). Deep learning–based automated detection algorithm for active pulmonary tuberculosis on chest radiographs: Diagnostic performance in systematic screening of asymptomatic individuals.. Eur. Radiol..

[31] Segato A., Marzullo A., Calimeri F., De Momi E. (2020). Artificial intelligence for brain diseases: A systematic review.. APL Bioeng..

[32] Naidoo J., Shelmerdine S.C., -Charcape C.F.U., Sodhi A.S. (2023). Artificial intelligence in *Paediatric tuberculosis*.. Pediatr. Radiol..

[33] Salau A.O., Jain S. (2021). Adaptive diagnostic machine learning technique for classification of cell decisions for AKT protein.. Inform. Med. Unlocked.

[34] Sara Z.S.L. Chapter 46 literature review of deep learning for tuberculosis based on chest imaging.. International Conference on Advanced Intelligent Systems for Sustainable Development.

[35] Suárez I., Fünger S. M., Rademacher J., Fätkenheuer G., Kröger S., Rybniker J. (2019). Diagnosis and therapy of tuberculosis.

[36] Melendez J., van Ginneken B., Maduskar P., Philipsen R.H.H.M., Ayles H., Sanchez C.I. (2016). On combining multiple-instance learning and active learning for computer-aided detection of tuberculosis.. IEEE Trans. Med. Imaging.

[37] Talat A., Khan A.U. (2023). Artificial intelligence as a smart approach to develop antimicrobial drug molecules: A paradigm to combat drug-resistant infections.. Drug Discov. Today.

[38] Orjuela-Cañón A.D., Jutinico A.L., Awad C., Vergara E., Palencia A. (2022). Machine learning in the loop for tuberculosis diagnosis support.. Front. Public Health.

[39] Zaman K. (2010). Tuberculosis: A global health problem.. J. Health Popul. Nutr..

[40] K.-M M., Murray J.A.N.J.F. (2016). Tuberculosis.. Textbook of Respiratory Medicine..

[41] Zhan Y., Wang Y., Zhang W., Ying B., Wang C. (2022). Diagnostic accuracy of the artificial intelligence methods in medical imaging for pulmonary tuberculosis: A systematic review and meta-analysis.. J. Clin. Med..

[42] Albuquerque V.V.S., Kumar N.P., Fukutani K.F., Vasconcelos B., Arriaga M.B., Silveira-Mattos P.S., Babu S., Andrade B.B. (2019). Plasma levels of C-reactive protein, matrix metalloproteinase-7 and lipopolysaccharide-binding protein distinguish active pulmonary or extrapulmonary tuberculosis from uninfected controls in children.. Cytokine.

[43] Hu X., Wang J., Ju Y., Zhang X., Qimanguli W., Li C., Yue L., Tuohetaerbaike B., Li Y., Wen H., Zhang W., Chen C., Yang Y., Wang J., Chen F., X. et al Hu (2022). Combining metabolome and clinical indicators with machine learning provides some promising diagnostic markers to precisely detect smear-positive/negative pulmonary tuberculosis.. BMC Infect. Dis..

[44] Ahmad R., Xie L., Pyle M., Suarez M.F., Broger T., Steinberg D., Ame S.M., Lucero M.G., Szucs M.J., MacMullan M., Berven F.S., Dutta A., Sanvictores D.M., Tallo V.L., Bencher R., Eisinger D.P., Dhingra U., Deb S., Ali S.M., Mehta S., Fawzi W.W., Riley I.D., Sazawal S., Premji Z., Black R., Murray C.J.L., Rodriguez B., Carr S.A., Walt D.R., Gillette M.A. (2019). A rapid triage test for active pulmonary tuberculosis in adult patients with persistent cough.. Sci. Transl. Med..

[45] Rahman T., Khandakar A., Kadir M.A., Islam K.R., Islam K.F., Mazhar R., Hamid T., Islam M.T., Kashem S., Mahbub Z.B., Ayari M.A., Chowdhury M.E.H. (2020). Reliable tuberculosis detection using chest X-ray with deep learning, segmentation, and visualization.. IEEE Access.

[46] (2020). Belarus tuberculosis portal.. B. P. Health..

[47] Jaeger S., Candemir S., Antani S., Wáng Y-X.J., Lu P-X., Thoma G. (2014). Two public chest X-ray datasets for computer-aided screening of pulmonary diseases.. Quant. Imaging Med. Surg..

[48] (2014). RSNA pneumonia detection challenge.. https://www.kaggle.com/competitions/rsna-pneumonia-detection-challenge.

[49] Bhardawaj F., Jain S. (2024). CAD system design for two-class brain tumor classification using transfer learning.. Curr. Cancer Ther. Rev..

[50] Gargya S., Jain S. (2023). CAD system design for pituitary tumor classification based on transfer learning technique.. Curr. Med. Imaging.

[51] Salau AO, Jain S Feature extraction: A survey of the types, techniques and applications.. 5th International Conference on Signal Processing and Communication (ICSC-2019).

[52] Rahman M., Cao Y., Sun X. (2021). Deep pre-trained networks as a feature extractor with XGBoost to detect tuberculosis from chest Xray.. Comput. Electr. Eng..

[53] Ahmad W.S.H.M.W, Ahmad M.F Comparison of different feature extraction techniques in content-based image retrieval for CT brain images.. 2008 IEEE 10th Workshop on Multimedia Signal Processing.

[54] Kumar R., Kumbharkar P., Vanam S., Sharma S. (2023). Medical images classification using deep learning: A survey.. Multimedia Tools Appl..

[55] Ortega J. A. R. (2011). Study of segmentation and identification techniques applied to environments with natural illumination and moving objects.. Polytechnic University of Valencia.

[56] Ansari M.A., Kurchaniya D., Dixit M. (2017). A comprehensive analysis of image edge detection techniques.. Int. J. Multimed. Ubiquitous Eng..

[57] Dharampal (2015). Methods of image edge detection.. J Electr Electron Syst.

[58] Rashmi Salavi, Mandar Sohani Textural feature based image classification using artificial neural network.. Advances in Computing, Communication and Control. ICAC3 2011..

[59] Tian D.P. (2013). A review on image feature extraction and representation techniques.. Int. J. Multimed. Ubiquitous Eng..

[60] Prashar N., Sood M., Jain S. (2020). Novel cardiac arrhythmia processing using machine learning techniques.. Int. J. Image Graph..

[61] Damljanovic A., Ruospo A., Sanchez E., Squillero G. (2022). Machine learning for hardware security: Classifier-based identification of Trojans in pipelined microprocessors.. Appl. Soft Comput..

[62] Singh M. (2022). Evolution of machine learning in tuberculosis diagnosis: A review of deep learning-based medical applications.. Electronics.

[63] Pal B., Jain S. (2022). Novel discrete component wavelet transform for detection of cerebrovascular diseases.. Sadhana.

[64] Jamwal A., Jain S. (2022). Robust multimodal fusion network employing novel empirical riglit wavelet transform for brain images.. Meas. Sensors.

[65] Theodosiou A.A., Read R.C. (2023). Artificial intelligence, machine learning and deep learning: Potential resources for the infection clinician.. J. Infect..

[66] Balakrishnan V., Kherabi Y., Ramanathan G., Paul S.A., Tiong C.K. (2023). Machine learning approaches in diagnosing tuberculosis through biomarkers - A systematic review.. Prog. Biophys. Mol. Biol..

[67] Pasa F., Golkov V., Pfeiffer F., Cremers D., Pfeiffer D. (2019). Efficient deep network architectures for fast chest x-ray tuberculosis screening and visualization.. Sci. Rep..

[68] Jain S., Salau A.O. (2023). Robust predictive model for different cancers due to biomarker proteins.. Curr. Signal Transduct. Ther..

[69] Helm J.M., Swiergosz A.M., Haeberle H.S., Karnuta J.M., Schaffer J.L., Krebs V.E., Spitzer A.I., Ramkumar P.N. (2020). Machine learning and artificial intelligence: Definitions, applications, and future directions.. Curr. Rev. Musculoskelet. Med..

[70] Perea-Jacobo R., Paredes-Gutiérrez G.R., Guerrero-Chevannier M.Á., Flores D.L., Muñiz-Salazar R. (2023). Machine learning of the whole genome sequence of *Mycobacterium tuberculosis*: A scoping prisma-based review.. Microorganisms.

[71] Tiwari S.M.A. Machine learning techniques for tuberculosis prediction.. International Conference on Advances in Engineering Science Management and Technology.

[72] Liang S. (2022). The application of artificial intelligence in the diagnosis and drug resistance prediction of Pulmonary tuberculosis.. Front. Med..

[73] Bhattamisra S. K., Banerjee P., Gupta P., Mayuren J., Patra S., Candasamy M. (2023). Artificial intelligence in pharmaceutical and healthcare research.. Big Data Cogn. Comput..

[74] Dohál M. (2023). Advancing tuberculosis management: The role of predictive, preventive, and personalized medicine.. Front. Microbiol..

[75] (2014). Tuberculosis Chest X-rays (Shenzhen).. https://www.kaggle.com/datasets/raddar/tuberculosis-chest-xrays-shenzhen.

